# Cancer-Associated circRNA–miRNA–mRNA Regulatory Networks: A Meta-Analysis

**DOI:** 10.3389/fmolb.2021.671309

**Published:** 2021-05-12

**Authors:** Shaheerah Khan, Atimukta Jha, Amaresh C. Panda, Anshuman Dixit

**Affiliations:** ^1^Institute of Life Sciences, Bhubaneswar, India; ^2^Regional Centre for Biotechnology, Faridabad, India; ^3^Manipal Academy of Higher Education, Manipal, India

**Keywords:** circRNA, microRNA, cancer, TCGA, driver genes, tumor suppressor genes

## Abstract

Recent advances in sequencing technologies and the discovery of non-coding RNAs (ncRNAs) have provided new insights in the molecular pathogenesis of cancers. Several studies have implicated the role of ncRNAs, including microRNAs (miRNAs), long non-coding RNAs (lncRNAs), and recently discovered circular RNAs (circRNAs) in tumorigenesis and metastasis. Unlike linear RNAs, circRNAs are highly stable and closed-loop RNA molecules. It has been established that circRNAs regulate gene expression by controlling the functions of miRNAs and RNA-binding protein (RBP) or by translating into proteins. The circRNA–miRNA–mRNA regulatory axis is associated with human diseases, such as cancers, Alzheimer’s disease, and diabetes. In this study, we explored the interaction among circRNAs, miRNAs, and their target genes in various cancers using state-of-the-art bioinformatics tools. We identified differentially expressed circRNAs, miRNAs, and mRNAs on multiple cancers from publicly available data. Furthermore, we identified many crucial drivers and tumor suppressor genes in the circRNA–miRNA–mRNA regulatory axis in various cancers. Together, this study data provide a deeper understanding of the circRNA–miRNA–mRNA regulatory mechanisms in cancers.

## Introduction

The cellular processes governing gene expression regulation are controlled at the molecular level. The aberrations in these regulations are linked to severe consequences, including cancers. Unfortunately, the biological processes pivotal to cancer growth and metastasis remain undefined despite the years of dedicated research. As a result, there is a dearth of molecular targets for diagnostics, prognostics, and therapeutics for many cancers. Improved insights into the molecular mechanisms of cancer development and progression will help develop the strategies for early diagnosis, prognosis, and treatment.

Recent developments in the high-throughput sequencing technologies led to the discovery of novel therapeutic biomolecules, including microRNAs (miRNAs), long non-coding RNAs (lncRNAs), and poorly characterized circular RNAs (circRNAs) (Guria et al., 2019). The miRNAs and lncRNAs are well known for their critical role in cancer development and metastasis (Huang et al., 2013; Slack and Chinnaiyan, 2019). In recent years, an increasing number of researchers are focusing their efforts on explaining the biological functions of circRNAs. The circRNAs, also known as competitive endogenous RNAs (ceRNAs), are a large family of covalently closed single-stranded stable RNA molecules with a regulatory potential (Salzman et al., 2012; Ledford, 2013). They are generated from exons and/or introns with a certain degree of evolutionary conservation and show tissue-specific expression patterns (Chen, 2016). Although the biological functions of the majority of circRNAs are not known, the accumulating pieces of evidence established that circRNAs regulate gene expression by sponging miRNAs and binding with RNA-binding proteins (RBPs), and by direct translation into proteins (Panda, 2018; Huang et al., 2020).

Circular RNAs are widespread; however, their expression is tissue-specific. A growing body of research indicates that circRNAs are involved in various types of pathophysiology, including aging, diabetes, glycolysis (Mirzaei and Hamblin, 2020), myogenesis (Das et al., 2020), virus infections (Nahand et al., 2020), and cancer (Haque and Harries, 2017; Cai et al., 2019; Shabaninejad et al., 2019; Shang et al., 2019; Borran et al., 2020; Li et al., 2020; Razavi et al., 2021). Since circRNAs are stable and tissue-specific, many studies have explored their diagnostic and prognostic potential in cancers. The emerging evidence indicates that circRNA can be an excellent biomarker for the development of new diagnostic and prognostic strategies. For instance, CDR1as and circ-FOXO3 are involved in regulating the development of breast cancer by acting as miRNA sponges (Lu, 2017; Yang et al., 2019). Furthermore, circ-ITCH inhibits the Wnt/β-catenin pathway in esophageal squamous cell carcinoma by sponging miRNAs (Li et al., 2015). Several studies suggested that the circRNA–miRNA–mRNA axis plays a crucial role in regulating various cellular events critical for cancer progression. However, the molecular mechanisms of circRNA–miRNA–mRNA regulatory axis in the carcinogenesis and progression of cancer are not well studied.

Understanding the circRNA–miRNA interaction can give important clues about the molecular mechanism of the pathogenesis in a given cancer. In the current study, the expression profiles of circRNAs, miRNAs, and mRNAs in different cancers have been collected from Gene Expression Omnibus (GEO) database, The Cancer Genome Atlas (TCGA), and research publications. The circRNA–miRNA–mRNA regulatory networks consisting of the differentially expressed (DE) circRNAs and their downstream miRNAs and target mRNAs have been constructed for seven cancer types. The circRNAs that may play active roles in regulating the driver genes and tumor suppressor genes in those cancers are also identified. The analysis of target mRNAs for the functional pathways using the protein–protein interaction network (PPIN) and gene ontology (GO) enrichment analysis revealed the potential mechanism of circRNAs in the initiation and progression of various cancers. Together, this research provides new insights into the regulation of carcinogenesis by the circRNA–miRNA–mRNA regulatory axis.

## Results

### Identification of DE circRNAs in Different Cancers

**FIGURE 1:**
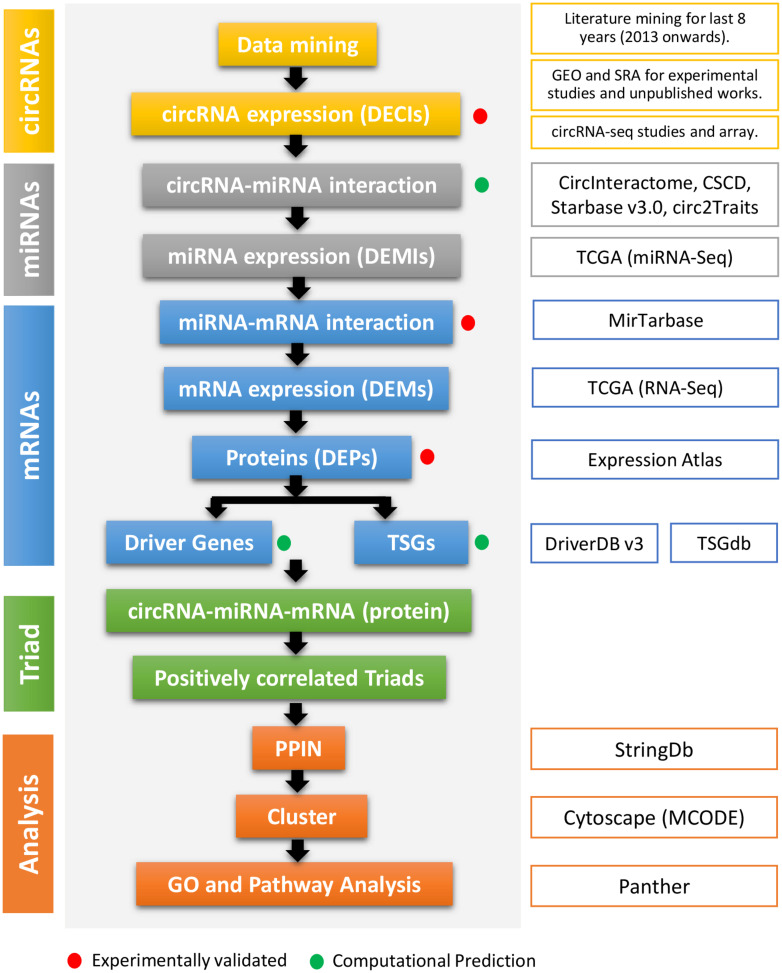
The flowchart of the method used in this study. The first column represents the types of regulatory elements (color-coded). The second column mentions the method cascade. The third column gives the detail about every step, especially the source of the study. The red and green dots represent if the step and output are experimentally validated or computationally predicted.

The data mining to identify DE circRNAs (DECIs) in different cancers resulted in more than 1,300 articles (i.e., research and review articles). Only research articles for the last 8 years were considered to find circRNAs associated with cancer (Figure 1 and Supplementary Table 1). The supplementary data from the reports were analyzed for DECIs, especially for those having a significant expression (*p* value < 0.05). The results from different circRNA databases were also included. The data were compiled such that all the circRNAs had at least a circBase ID and genomic coordinates. Other relevant information such as the gene symbol, type of circRNA (exonic/intronic), regulation (up- or downregulated), and strand were also included wherever available. These DECIs were classified into different types such as exonic, intronic, intergenic, intragenic, sense overlapping, and antisense circRNAs (Supplementary Table 2).

### Identification of circRNA–miRNA and miRNA–mRNA Interactions and Construction of the ceRNA Network

The expression of the mRNAs is tightly controlled at the posttranscriptional level by RBPs and miRNAs. The miRNAs can regulate the expression of mRNAs by promoting their 3′ degradation, whereas the circRNAs can exert their control by acting as a miRNA sponge and thereby controlling mRNA regulation indirectly. Understanding the circRNA–miRNA and miRNA–mRNA interactions can give important clues about the molecular mechanism of the pathogenesis in a given cancer. The *in silico* prediction algorithms were utilized to produce the circRNA–miRNA and miRNA–mRNA interaction map.

**FIGURE 2:**
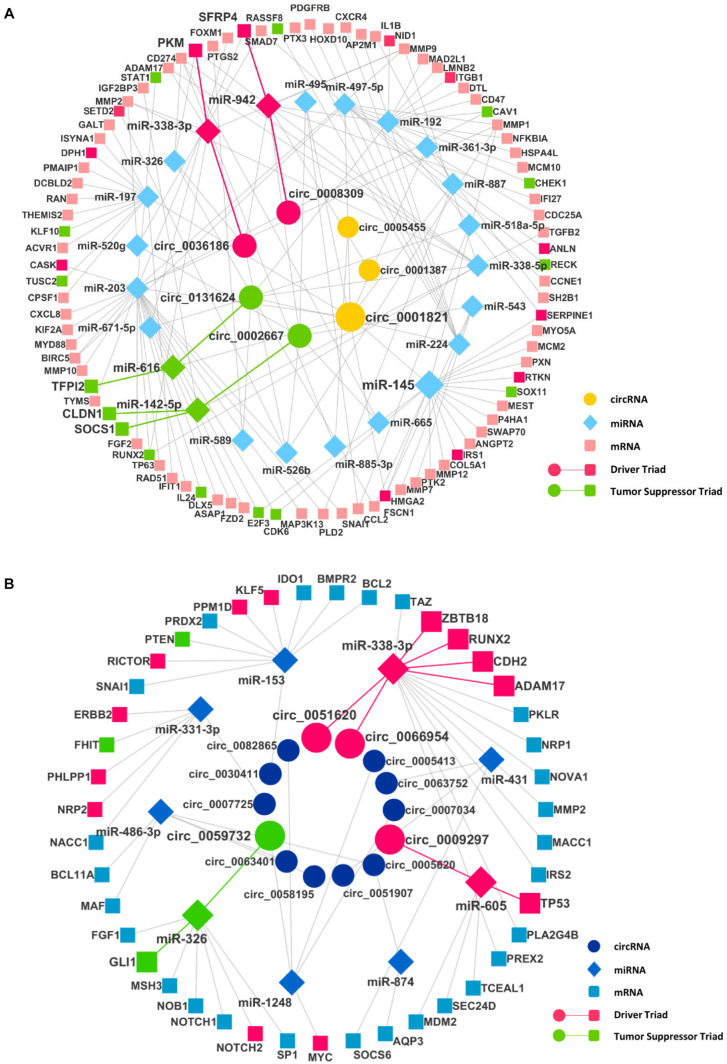
The circRNA–miRNA–mRNA regulatory networks in **(A)** head and neck squamous cell carcinoma (HNSCC) and **(B)** lung cancer. Nodes (inner to outer) represent differentially expressed circRNAs, miRNAs, and mRNAs, respectively. The circular nodes represent circRNA, diamonds represent miRNAs, and square nodes represent the mRNAs. The red and the green highlighted circRNA–miRNA–mRNA interaction represent a driver and tumor suppressor triads, respectively.

After finding the DECIs, the next step was to find the circRNA–miRNA interaction. The miRNAs that have two or more binding sites on circRNAs were considered only to create the circRNA–miRNA interaction. The analysis of these miRNAs in mirTarBase resulted in the identification of many mRNAs as targets, resulting in the miRNA–mRNA interaction. To identify significantly DE miRNAs (DEMIs) and mRNAs, we analyzed transcriptomics data in TCGA for seven different cancers. We included only DEMI and DE mRNA (DEM) in chosen cancers for this study. Hence, the number of circRNA–miRNA and miRNA–mRNA interactions were reduced (Supplementary Table 3). Furthermore, these interactions were merged to form a circRNA–miRNA–mRNA triad (Figure 2). The identified triad with a positive correlation between the circRNA and mRNA expression was only considered further. A network of triads was generated to understand their interrelation and possible role in the pathogenesis of cancer. Besides, we also scrutinized the presence of tumor driver and suppressor genes in DEMs. The circRNAs, hsa_circ_0036186| PKM2, are known to regulate 14-3-3-ζ expression by functioning as a ceRNA in the development and progression of head and neck squamous cell carcinoma (HNSCC) (Li B. et al., 2018). It is important to note that SFRP4, a driver gene, upregulated in HNSCC, is regulated by five different circRNAs, namely, hsa_circ_0008309| CUL3, hsa_circ_0001387| WHSC1, hsa_circ_0036186| PKM2, hsa_circ_0002667| MGAT2, and hsa_circ_0001821| circPVT1, in the identified triad. SFRP4, which drives the process of carcinogenesis in HNSCC, has not been reported anywhere about its interaction with circWHSC1. In this study, it is seen to be regulated by the circRNA hsa_circ_0001387| WHSC1 through miR-942. Similarly, in lung cancer, the circRNAs, namely, hsa_circ_0051620| SLC1A5 and hsa_circ_0066954| POLQ, are upregulated and are shown to interact with and regulate driver genes, namely, ADAM17, CDH2, RUNX2, and ZBTB18, through miR-338-3p. The miRNA-338-3p, however, is known to suppress tumor proliferation (Ni et al., 2013). Although circRNA function is not yet understood completely, the circRNA–miRNA–mRNA network analysis suggests that these circRNAs may act as a miRNA sponge and regulate the driver genes, thereby modulating carcinogenesis. The driver and tumor suppressor genes regulated by the miRNA and circRNA were marked in the network as driver and tumor suppressor triads, respectively (Supplementary Table 4). We now have a DE triad of circRNA–miRNA–mRNA for seven different cancers (Supplementary Table 5). A circRNA–miRNA–mRNA triad network was made for each cancer (Supplementary Material).

### Construction of PPIN and Extraction of Clusters

**FIGURE 3:**
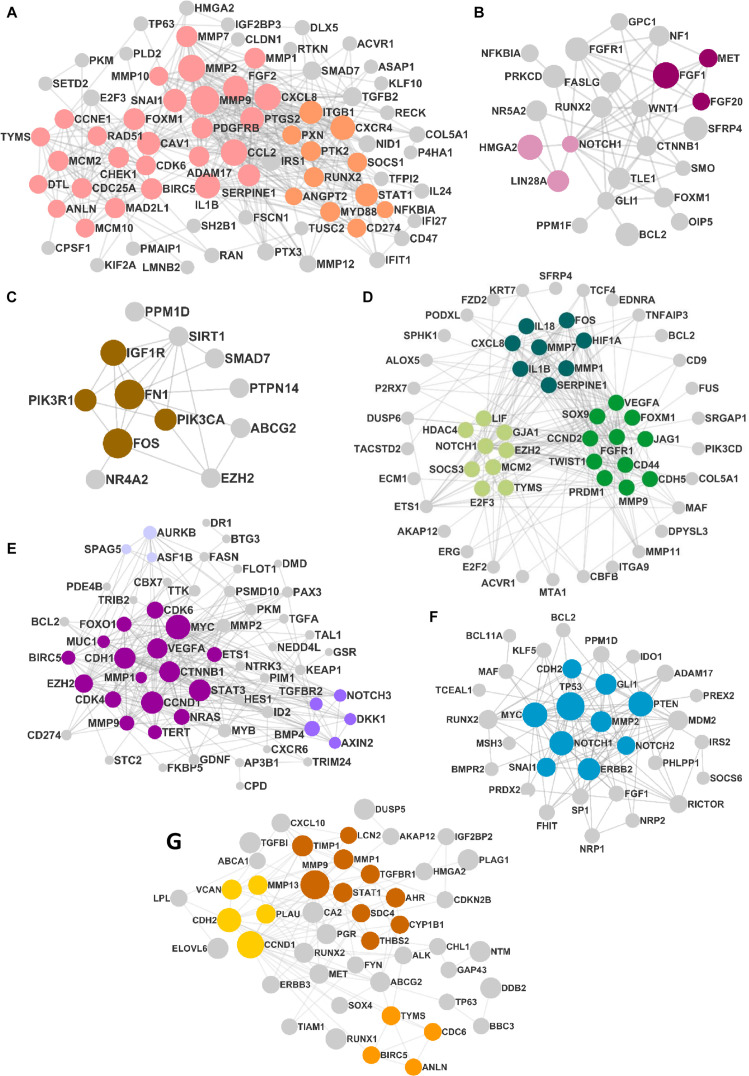
The protein–protein interaction network (PPIN) in **(A)** head and neck squamous cell carcinoma (HNSCC), **(B)** breast cancer, **(C)** pancreatic cancer, **(D)** gastric cancer, **(E)** liver cancer, **(F)** lung cancer, and **(G)** thyroid cancer. The size of the nodes is distributed according to the number of in and out degrees.

**FIGURE 4:**
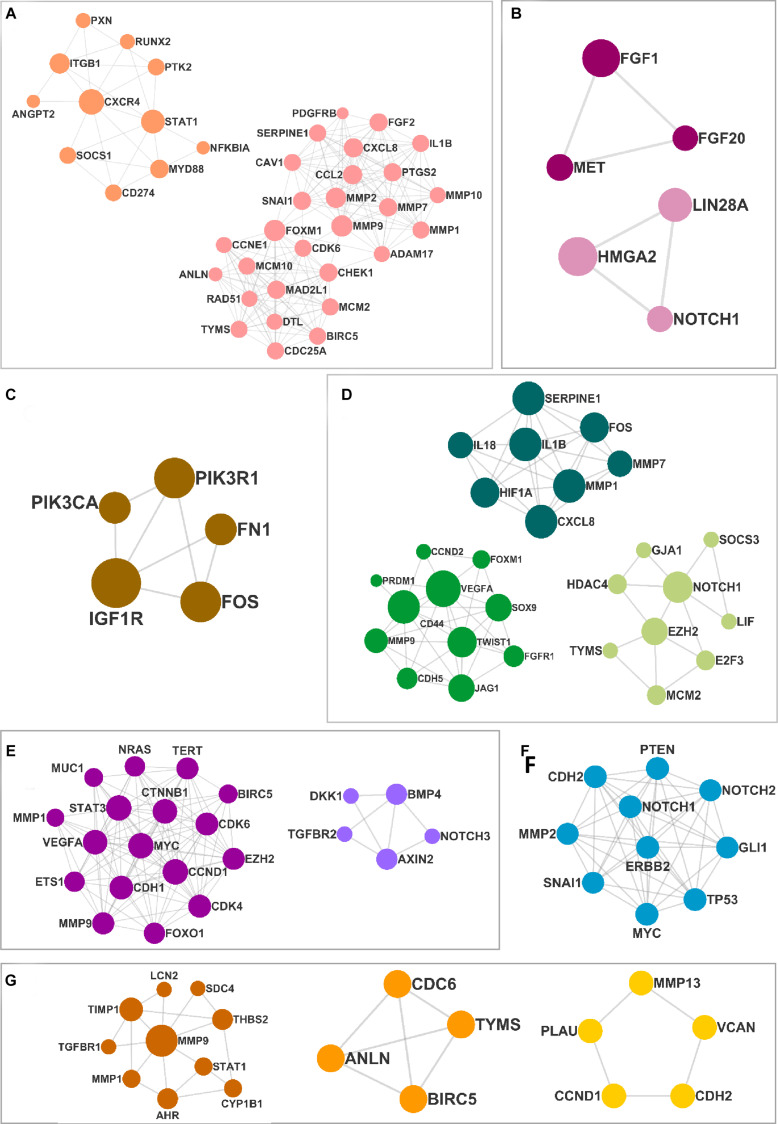
The clusters extracted from the protein–protein interaction network (PPIN) for **(A)** head and neck squamous cell carcinoma (HNSCC), **(B)** breast cancer, **(C)** pancreatic cancer, **(D)** gastric cancer, **(E)** liver cancer, **(F)** lung cancer, and **(G)** thyroid cancer.

The DEMs from the circRNA–miRNA–mRNA triads were used to create the PPIN in the form of a network graph for each cancer (Figure 3). The analysis of a large PPIN can give information about small subnetworks, also known as “clusters.” In this study, the advantage of the clusters in a PPIN is to get information about specific processes represented by them. The modulation of any protein expression in the PPIN by the circRNA–miRNA–mRNA networks may affect the function of the cluster and the pathway. This analysis can also give information about the hub genes that may be important for the stability and functioning of a given PPIN. In this study, the MCODE algorithm was used to extract clusters from the generated PPIN (Figure 4). The PPIN for different cancers resulted in different number of clusters (Supplementary Table 6).

### GO and Pathway Analysis

**FIGURE 5:**
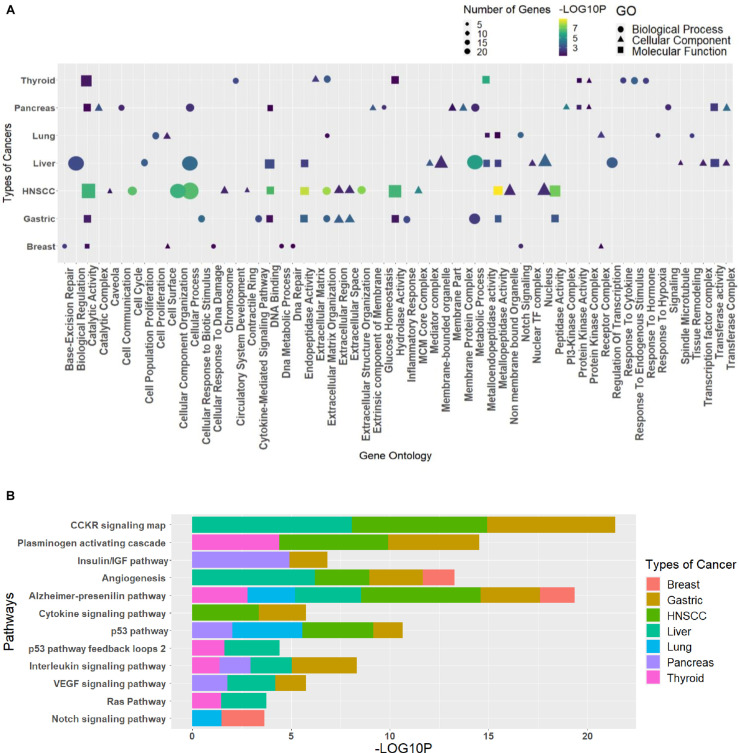
The topmost common **(A)** gene ontologies and **(B)** pathways, taken from the largest cluster of each cancer. The graph has been plotted based on the significant *p* values (*p* ≤ 0.05), taken as the negative logarithm of the *p* value (–log_10_*p*).

The GO analysis for the biological process, molecular function, and cellular component can give important information about the processes and pathways in which a group of genes may be involved. Such information is vital for the generation of hypotheses and the design of further studies. The enriched GO terms and pathways for different clusters were analyzed to see the pathways and processes in which they are involved (Supplementary Table 7). Every cluster has its significance in terms of functions; hence, the need to classify each cluster differently in terms of their processes helps us relate these essential processes to the circRNAs indirectly through the triad. The R package ggplot2 was used to plot the graphs, where GO was combined in dot plots and pathways as bar plots (Supplementary Material). The most common processes among cancers were extracellular matrix organization (GO:0030198), cellular process (GO:0009987), metabolic process (GO:0008152), catalytic activity (GO:0003824), metallopeptidase activity (GO:0008237), hydrolase activity (GO:0016787), extracellular region (GO:0005576), and nucleus (GO:0005634), and the pathways were Alzheimer’s disease–presenilin pathway (P00004), p53 pathway (P00059), and angiogenesis (P00005). The topmost common gene ontologies and pathways from every largest cluster for each cancer are taken and plotted (Figure 5).

### Impact of DEMIs and DEMs on Patient Survival

**FIGURE 6:**
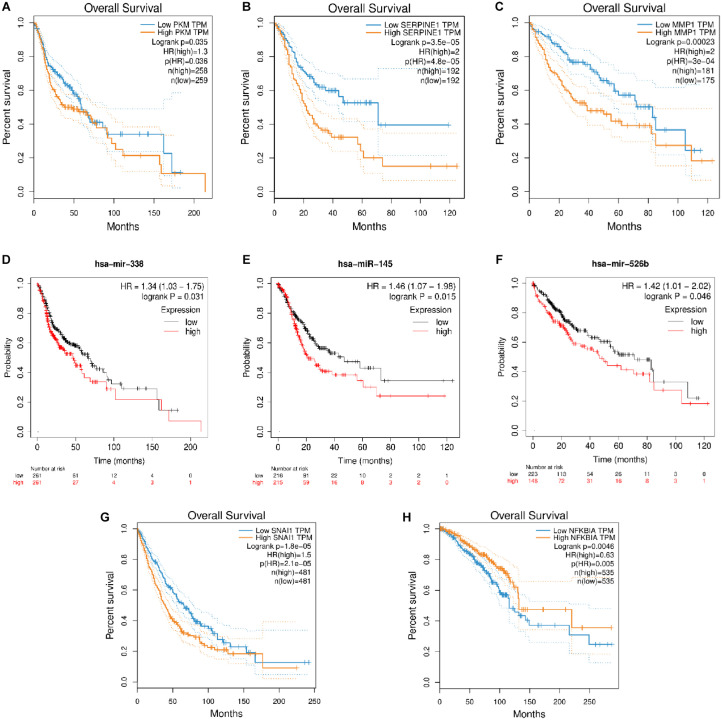
The survival plots for **(A)** PKM and its corresponding miRNA, **(D)** has-miR-338-5p in HNSCC, **(B)** SERPINE1 and its corresponding miRNA, **(E)** hsa-miR-145 in gastric cancer, **(C)** MMP1 and its corresponding miRNA, **(F)** hsa-miR-526b in liver cancer, **(G)** SNAI1 in lung cancer and **(H)** NFKBIA in breast cancer.

The role of many coding RNAs and non-coding RNAs (ncRNAs) has been studied in various cancers to determine their impact on the survival of the patients. Besides mRNA, there are many ncRNAs, such as miRNAs (onco–micro RNAs), whose expression has been seen to affect the survival of the patients. Several DEMs and DEMIs from the triads were common, with the top DE genes and miRNAs playing a significant role in the overall survival of patients (Supplementary Material). The circRNAs interacting with those DEMIs and DEMs can be predicted to have similar functions. For example, the circRNAs in a triad hsa_circ_0131624| TUBB2A–hsa-miR-338-5p—PKM in HNSCC, hsa_circ_0080517| CLDN4–hsa-miR-145—SERPINE1 in gastric cancer, and hsa_circ_0000228| ZEB1–hsa-miR-526b—MMP1 and hsa_circ_0009022| PPP4R1–hsa-miR-526b—MMP1 in liver cancer, where both the miRNAs and mRNAs from the triad play a significant role in survival, might be important in the prognosis of these cancers. Hence, we plotted the survival curves for HNSCC, gastric cancer, liver cancer, lung cancer, and breast cancer using DEMs and DEMIs in triads (Figure 6). We did not find any significant DEMs and DEMIs in our triads for pancreatic cancer and thyroid cancer, affecting the overall survival (Supplementary Table 8).

## Materials and Methods

### Data Mining

PubMed was used to collect the circRNAs associated with 15 different cancers (Supplementary Table 1). The search was performed using the keywords “name of cancer” and “circular RNA,” for example, “HNSCC” or “head and neck squamous,” “cancer” or “tumor,” and “tumor” or “carcinoma,” and “circRNA” or “ciRNA” or “Circular RNA” were used for HNSCC. The results were further filtered using the following inclusion criteria: (i) research articles for the last 7 years (2013 onward), (ii) studies done exclusively on humans, (iii) circRNAs with the expression as | Log 2 Fold Change ≥ 1| and *p* value (<0.05). The review articles, redundant papers (update of a previous work done by the same authors), and circRNAs having ArrayStar Id without genomic coordinates were excluded.

Moreover, the NCBI GEO was explored for the unpublished experimental work, including the microarray and total RNAseq datasets for the role of circRNAs and their expression in the selected cancers. The studies containing circRNA expression data for at least three replicates (tumor and non-tumor) were chosen for further analysis. Additionally, other circRNA databases such as CircR2Disease (Fan et al., 2018), CircFunBase (Meng et al., 2019), CircInteractome (Dudekula et al., 2016), Circ2Traits (Ghosal et al., 2013), StarBase (Li et al., 2014), CircNet, CircBase (Liu et al., 2016), CIRCpedia (Dong et al., 2018), CSCD (Xia et al., 2018), TSCD (Xia et al., 2017), circRNADb (Chen et al., 2016), and ExoRbase (Li S. et al., 2018) were also searched. We considered only those circRNAs where the cancer type and expression data were available.

### Identification of circRNA–miRNA and miRNA–mRNA Interactions

To understand the circRNA–miRNA and miRNA–mRNA interactions, various bioinformatics tools, such as Cancer-Specific CircRNA (CSCD), CircInteractome, Circ2Traits, and StarBase, for each cancer were used. Additionally, to avoid bias by one algorithm, the circRNA–miRNA interaction identified by at least two algorithms was considered for further studies. The next part was to find the targets of the miRNAs identified in the previous step. For that, mirTarBase (Chou et al., 2018), a database that contains >360,000 experimentally validated miRNA–mRNA interactions (MTIs), was used. The target genes validated by at least more than one experimental method were selected for further analysis.

### Mining of DECIs, DEMIs, and DEMs

We identified DECIs, DEMIs, and DEMs using TCGA data (Cancer Genome Atlas Research Network [CGARN], Weinstein et al., 2013). The RNA-seq data for mRNAs and miRNAs for selected cancers were downloaded from TCGA and the differential expression analysis was performed using the DESEQ2 package (Love et al., 2014) in Bioconductor. A miRNA/mRNA was considered DE if | log2(fold change)| ≥ 1 and *p* value ≤ 0.05. Moreover, we also wanted to see if these DEMs are translated into proteins. We used Expression Atlas (Papatheodorou et al., 2018) to check if the DEMs are expressed in a given tissue for a particular cancer. We also considered mRNAs only if they are expressed as proteins in a particular cancer.

### Identification of Cancer Driver Genes

Although many mRNAs are DE during cancer development and progression, only a few are responsible for driving the disease process. These cancer driver genes give cells a growth advantage, especially when they are mutated or otherwise dysregulated. In this study, we used the DriverDBv3 (Jin et al., 2020) that uses 12 algorithms, such as ActiveDriver (Reimand and Bader, 2013), CoMET (Martin et al., 2015), Dendrix (Vandin et al., 2012), DriverML (Han et al., 2019), DriverNet (Bashashati et al., 2012), e-Driver (Porta-Pardo and Godzik, 2014), iPAC (Ryslik et al., 2013), MEMo (Ciriello et al., 2013), MSEA (Jia et al., 2014), MUTEX (Babur et al., 2015), NetBox (Cerami et al., 2010), and OncodriveCLUST (Tamborero et al., 2013), to predict the cancer driver genes.

### Identification of Tumor Suppressor Genes

Apart from the driver genes, another category is called anti-oncogene or tumor suppressor genes that help in cell growth regulation. These genes act like breaks in cell growth and multiplication. Mutations in tumor suppressor genes may also lead to cancer. Therefore, their study can identify essential mRNAs and, in turn, essential circRNAs regulating their activity. We used TSGene 2.0 (Zhao et al., 2016) to determine the tumor suppressor mRNAs. It contains 1,217 human TSGs (1,018 protein-coding and 199 non-coding genes) curated from more than 9,000 articles. Additionally, TSGene 2.0 provides thousands of expression and mutation patterns derived from TCGA.

### Generation of circRNA–miRNA–mRNA Triad

The circRNA–miRNA and miRNA–mRNA interactions were combined to form a circRNA–miRNA–mRNA regulatory triad. The next step was to identify high-priority triads based on the observed expression levels of coding and non-coding RNAs. As stated earlier, the circRNA may regulate the expression of mRNA by sponging the intermediate miRNAs. Thus, a positive correlation between the expression levels of circRNA and mRNA can be expected. It may indicate a strong effect of circRNA on the expression of given mRNA. A triad, in which circRNA is up and its corresponding mRNA(s) are also significantly up or circRNA is down and its corresponding mRNA(s) are also significantly down, is considered to be in a positive regulation. Therefore, we retained the triads if there exists a positive correlation between circRNA and mRNA expression levels.

### Construction and Analysis of PPIN

In the current study, the proteins related to the mRNAs from positive triads were used to create a PPIN for each cancer using the String database (Szklarczyk et al., 2016), where the minimum required interaction score was set at 0.4 (medium confidence). The PPIN was imported into Cytoscape v3.7.2 (Shannon et al., 2003) for further analysis. The highest interconnected component, also known as the “giant component,” was extracted. The clusters were then identified using MCODE (Bader and Hogu, 2003) algorithm that identifies densely connected nodes in a network. The layout was designed using the “combined score” for each protein combined with gene fusion, phylogenetic cooccurrence, homology, coexpression, experimental validation scores, and the node attribute.

### GO and Pathway Analysis

The gene ontologies and pathways were analyzed for the identified clusters using PANTHERv14.0 (Mi et al., 2019). The statistical overrepresentation test was used to find the enriched GO terms and pathways by matching the gene list with the human genome, applying Fisher’s exact test with Bonferroni correction. The GO and pathway analysis for the clusters relates the genes with specific processes and pathways. This information can be used to relate the circRNAs in the regulation of those processes and pathways. The R package, ggplot2 (Wickham, 2011), was used to plot the GO as dot plots and pathways as bar graphs based on the *p* values.

### Impact of DEMIs and DEMs on Patient Survival

We used GEPIA (Gene Expression Profiling Interactive Analysis) (Yang et al., 2020) for DEMs, and OncomiR (Wong et al., 2018) and UALCAN (Chandrashekar et al., 2017) for DEMIs, which considers the RNAseq and miRNAseq data from TCGA to see if the DEMs and DEMIs in our triad have a significant impact on survival. The Kaplan–Meier method (Nagy et al., 2018) was used to plot the overall survival curve for the DEMIs. The survival plots for both DEMs and DEMIs were considered significant, only if the log rank *p* value ≤ 0.05.

## Discussion

The advent of NGS technologies coupled with user-friendly tools has spurred research in deciphering the genome and its regulation. These technologies have arguably impacted the research in circRNA and have resulted in identifying many circRNAs with the myriads of functions. Since 2013, there has been a lot of attention given to circRNA research due to their novel functions such as miRNA sponging, RBP regulation, and translational capabilities. The circRNAs have sparked considerable interest as potential biomarkers due to their tissue-specific expression and high stability.

The circRNA–miRNA–mRNA axis plays a vital role in cancer initiation and progression (Jamal et al., 2019). For instance, the members of the circ-ZEB1 family are reported to play a role in the suppression of lung cancer progression *via* the sponging of miR-200. The circMOT1 sponges miR-9 to allow the expression of the tumor suppressor gene p21. The emerging oncogenic function of circRNAs is of particular interest, as it might make them candidates for new biomarkers and therapeutic targets in cancer. This study was made to find circRNAs in different cancers and group them into triads to see their role in controlling the downstream regulatory elements and their process.

This study has assimilated information from multiple sources such as literature search and online databases, including CircR2Disease, CircFunBase, CircInteractome, Circ2Traits, and StarBase TCGA, and GEO to establish a “Triad Regulatory Network.” The circRNA enhances gene expression by acting as miRNA sponges. This network was made based upon the established mechanism of interaction among circRNA, miRNA, and mRNA. If circRNA is overexpressed, it competitively binds to the miRNA and inhibits their activity, hence rescuing the mRNA degradation or *vice versa*. Therefore, we chose those triads with a change in the same direction for circRNAs and mRNA expression. We also established the driver and tumor suppressor triads based on the driver and tumor suppressor genes in our triad, followed by the GO and pathway analysis for the clusters. The hub genes (clusters), which were explicitly driver and tumor suppressor genes extracted from PPIN, were further analyzed for their therapeutic role(s).

In HNSCC, the driver gene PKM plays a vital role in carcinogenesis through cell proliferation. It is targeted by miR-338-3p, which in turn is targeted by hsa_circ_0036186| PKM2. As predicted in this study, this particular driver triad gains more importance as the survival study on the driver gene (PKM) and the miRNA that it interacts with (hsa-miR-338-3p) shows that both the gene and miRNA cause a decrease in the survival of the patients with HNSCC. One of the circRNAs, hsa_circ_0001387| WHSC1, predicted from this study and shown in our triad in HNSCC is upregulated and known to be circulating in the peripheral blood. This can act as a diagnostic marker for HNSCC.

In the lung cancer, the driver triad hsa_circ_0051620| SLC1A5–hsa-miR-338-3p—CDH2 and hsa_circ_0066954| POLQ–hsa-miR-338-3p—CDH2 shows the downregulation of the driver gene CDH2, which increases the survival of the patients. Since the circRNAs hsa_circ_0051620| SLC1A5 and hsa_circ_0066954| POLQ interact with CDH2, they might have a prognostic value in cancer.

We also found that most of the circRNAs among the seven different cancers are unique, except hsa_circ_0074817| EBF1, which was common between liver cancer and thyroid cancer and hsa_circ_0001821| circPVT1, which was common between head and neck cancer and gastric cancer. In liver cancer, hsa_circ_0074817| EBF1 targets miR-539-5p, targeting CDK4 and SPAG5. The upregulation of CDK4 triggers the development of non-alcoholic fatty liver disease (NFALD), which leads to the phosphorylation of C/EBPα on Ser193 and the formation of C/EBPα-p300 complexes, resulting in hepatic steatosis, fibrosis, and liver cancer. The overexpression of SPAG5 promoted tumor growth and metastasis, as SPAG5 interacts with CEP55 to trigger the phosphorylation of AKT at Ser473, causing liver cancer. In thyroid cancer, hsa_circ_0074817 targets miR-27a-3p, targeting MET, ABCA1, MMP13, and PLAG1, promoting cell proliferation, invasion, and metastasis in thyroid carcinoma. The circRNA hsa_circ_0001821| circPVT1 has common miRNA targets in HNSCC and gastric cancer, except miRNAs, i.e., hsa-miR-361-3p and hsa-miR-497-5p that are specific in HNSCC and hsa-miR-125 in gastric cancer.

This study acknowledges the fact that these findings are based on computational analysis and remain predictive until validated. Although we have tried to provide information about the mRNAs that are significantly translated into proteins (DEPs), yet the utilization of proteomics data might provide additional information to understand the behavior of the proteins in the regulatory triad. However, the strength of this study lies in the fact that it is the first that specifies the circRNA–miRNA–mRNA triad that might play a role in regulating the downstream process in different cancers based on (1) the positive and negative correlations among the regulatory elements, (2) mRNAs translated to proteins (DEPs), (3) the driver and tumor suppressor triads, and (4) the cluster-specific triads classifying the circRNAs into specific biological processes and functions. The future aspect would be to validate the circRNA–miRNA–mRNA axis and the possible functional roles experimentally. The circRNA sequencing from cell lines and patient samples would validate the *in silico* findings in this study and indicates the abundance of novel circRNAs. We also planned to find the function of circRNAs as miRNA sponges using various assays, including immunoprecipitation, miRNA pull-down assays, and luciferase activity analysis (Wu et al., 2018; Zhang et al., 2019; Lin et al., 2020). This study provides essential pathways enriched in different cancers. A study based on how the circRNAs influencing the cancer pathways, e.g., apoptosis, epithelial-mesenchymal transition (EMT) pathways, and angiogenesis, would help understand their roles in the pathogenesis. We also planned on working toward the development of circRNA-based therapeutic approaches (Holdt et al., 2018) by manipulating the circRNA expression, either knocking down (Wang et al., 2016) or overexpressing it (Zeng et al., 2017). Besides the understanding of circRNAs and work done in this field, which is still minimal at present, there are many other things about circRNA, including circRNA structure, degradation, biogenesis, and interaction with other RNAs, which remain undiscovered. This study is a step toward understanding the world of non-coding RNAs and their mechanisms which remain unexplored.

## Data Availability Statement

The original contributions presented in the study are included in the article/Supplementary Material, further inquiries can be directed to the corresponding author.

## Author Contributions

AD, AP, and SK conceptualized the study. SK and AJ performed the investigation and curated the data. AD and AP supervised the workflow. AD and SK prepared the original draft. All authors reviewed, edited, and approved the manuscript for publication.

## Conflict of Interest

The authors declare that the research was conducted in the absence of any commercial or financial relationships that could be construed as a potential conflict of interest.
