# Supplementary material for: Cancer-Associated circRNA–miRNA–mRNA Regulatory Networks: A Meta-Analysis
Source: Front Mol Biosci. 2021 May 12;8:671309. doi: 10.3389/fmolb.2021.671309 (PMC8149909; doi:10.3389/fmolb.2021.671309)
Supplement: Supplementary file 9 [file Data_Sheet_1.docx]

Supplementary Material

Supplementary Figures


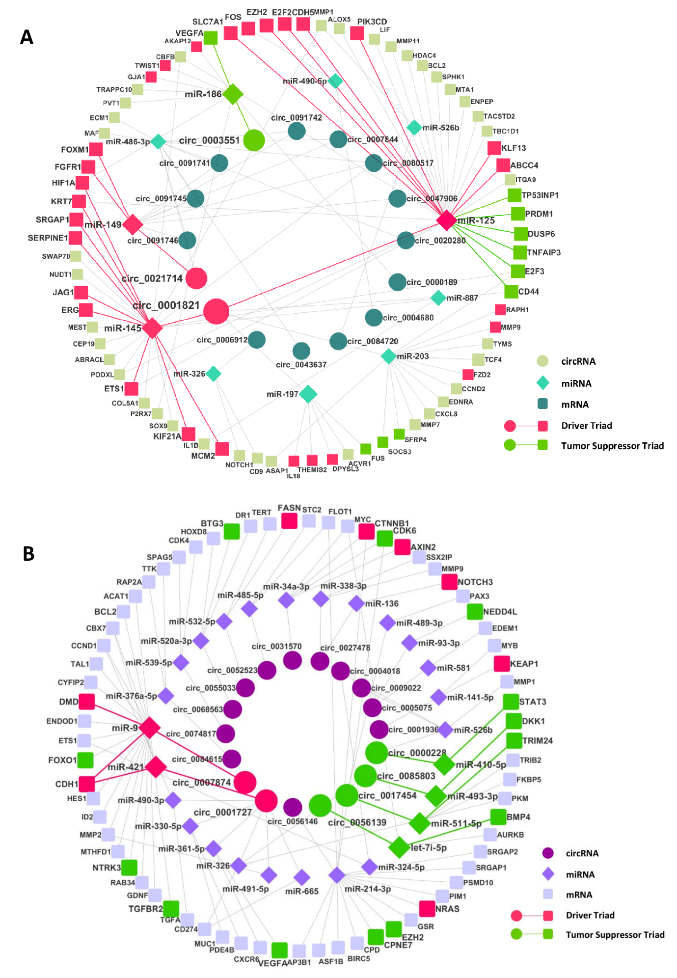

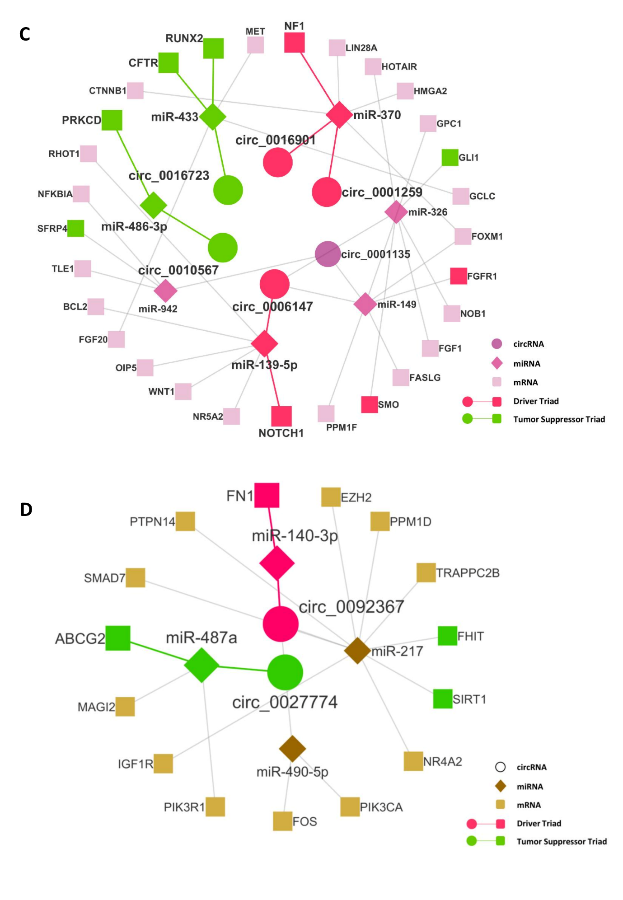

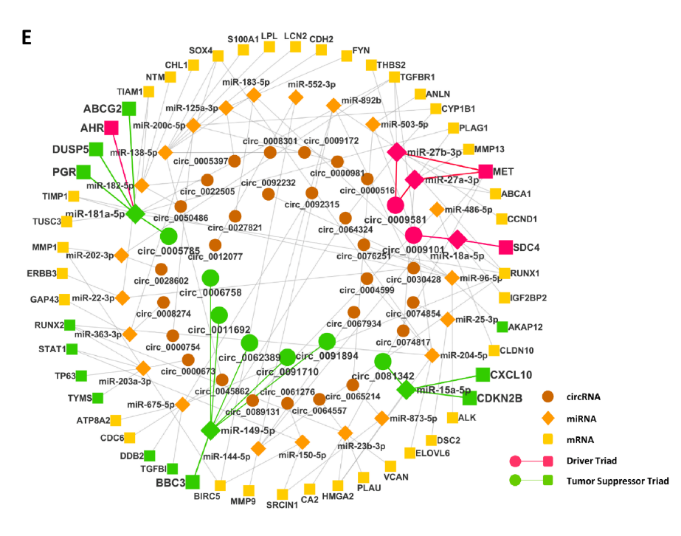


**Supplementary Figure 1.** The circRNA-miRNA-mRNA(protein) Regulatory network, in (A) gastric cancer, (B) liver cancer, (C) breast cancer, (D) pancreatic cancer, and (E) thyroid cancer. Nodes (inner to outer), represents differentially expressed circular RNAs, miRNAs and mRNAs, respectively. The circular nodes represent circular RNA, diamonds represent miRNAs and square nodes represent the mRNAs. The red and the green highlighted circRNA-miRNA-mRNA interaction represent driver and tumor suppressor triads, respectively.

**
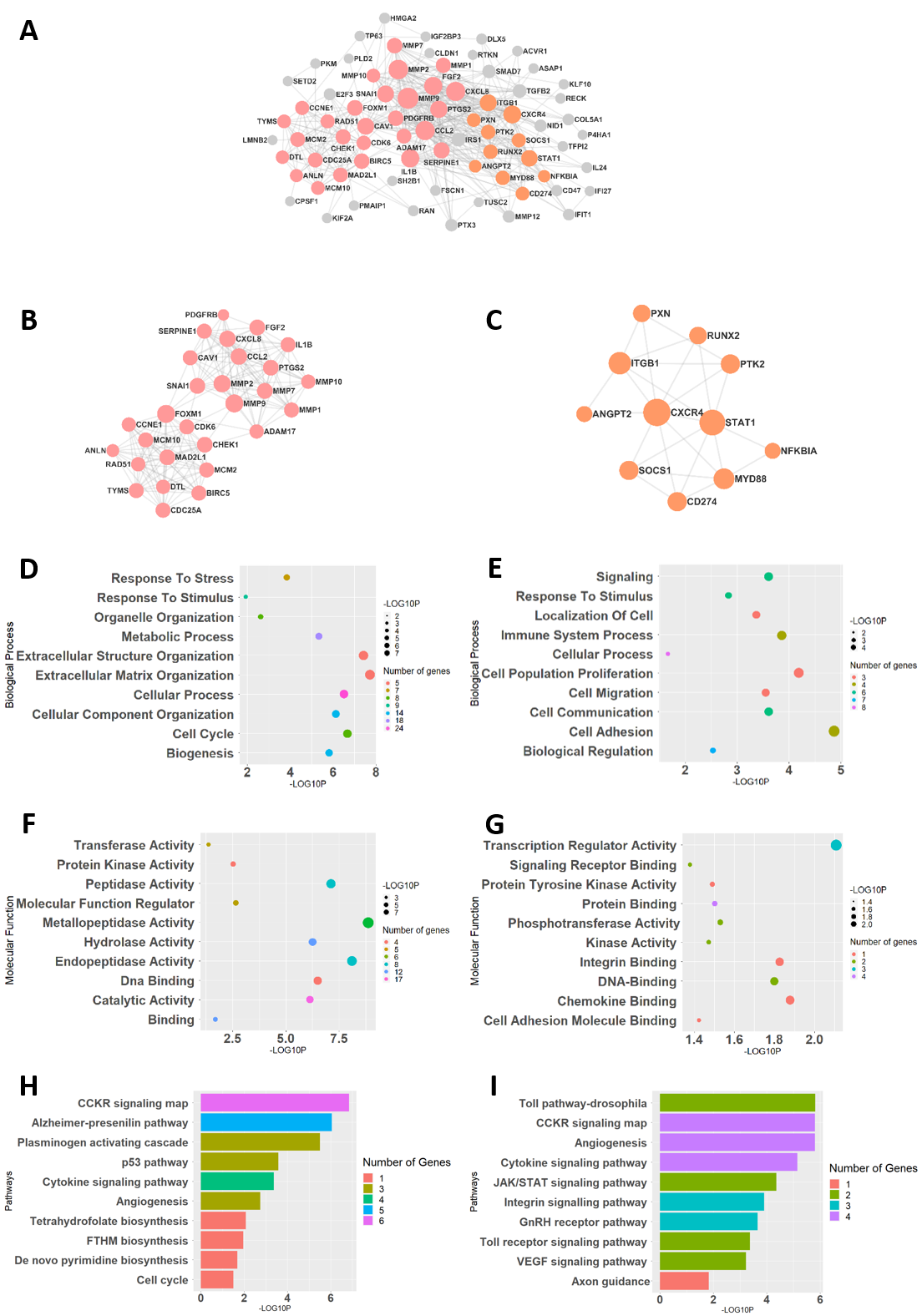
**

**Supplementary Figure 2.** (A) The Protein Protein Interaction Network (PPIN), in HNSCC. The clusters, (B) cluster 1 and (C) cluster 2, extracted from the Protein Protein Interaction Network (PPIN). The size of the nodes are distributed according to the number of in and out degrees. (D-I) The top 10 biological process, molecular function and pathways in HNSCC, for cluster 1 (D,F,H), cluster 2 (E,G,I), respectively. The graph has been plotted on the basis of the significant p values (<=0.05), taken as logarithm of p value (–log10P). The size of the dot is represented by the logarithm of the p value and the color represents the number of genes involved in a particular process.


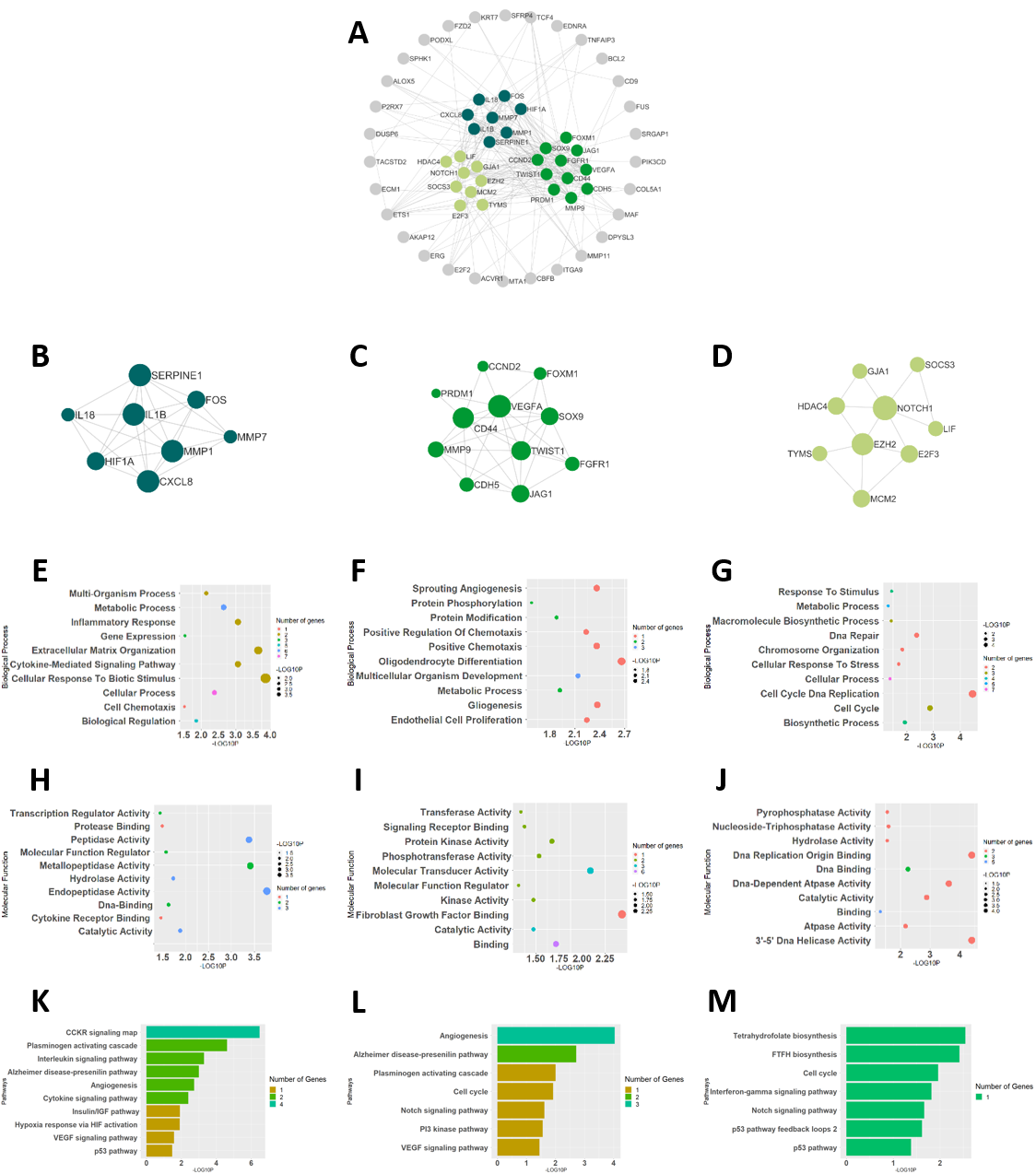


**Supplementary Figure 3**. (A) The Protein Protein Interaction Network (PPIN), in gastric cancer. (B-D) The clusters, cluster 1 (B), cluster 2 (C), and cluster 3 (D), extracted from the Protein Protein Interaction Network (PPIN), in gastric cancer. The size of the nodes are distributed according to the number of in and out degrees. (E-M) The top 10 biological process, molecular function and pathways in gastric cancer, for cluster 1 (E,H,K), cluster 2 (F,I,L), and cluster3 (G,J,M) respectively. The graph has been plotted on the basis of the significant p values (<=0.05), taken as logarithm of p value (–log10P). The size of the dot is represented by the logarithm of the p value and the color represents the number of genes involved in a particular process.


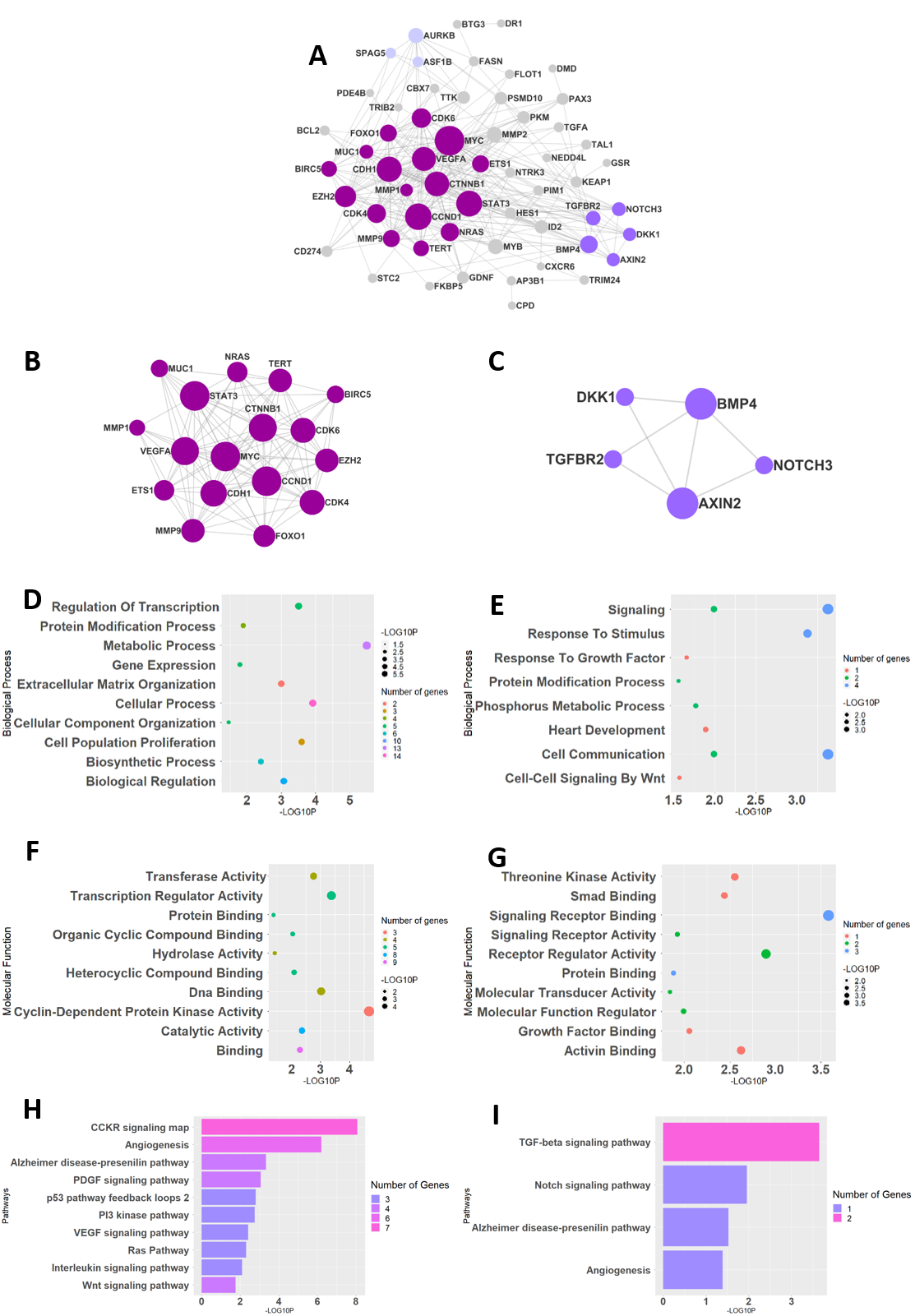


**Supplementary Figure 4**. (A) The Protein Protein Interaction Network (PPIN), in liver cancer. (B-C) The clusters, cluster 1 (B) and cluster 2 (C), extracted from the Protein Protein Interaction Network (PPIN), in liver cancer. The size of the nodes are distributed according to the number of in and out degrees. (D-I) : The top 10 biological process, molecular function and pathways in liver cancer, for cluster 1 (D,F,H), cluster 2 (E,G,I), respectively. The graph has been plotted on the basis of the significant p values (<=0.05), taken as logarithm of p value (–log10P). The size of the dot is represented by the logarithm of the p value and the color represents the number of genes involved in a particular process.


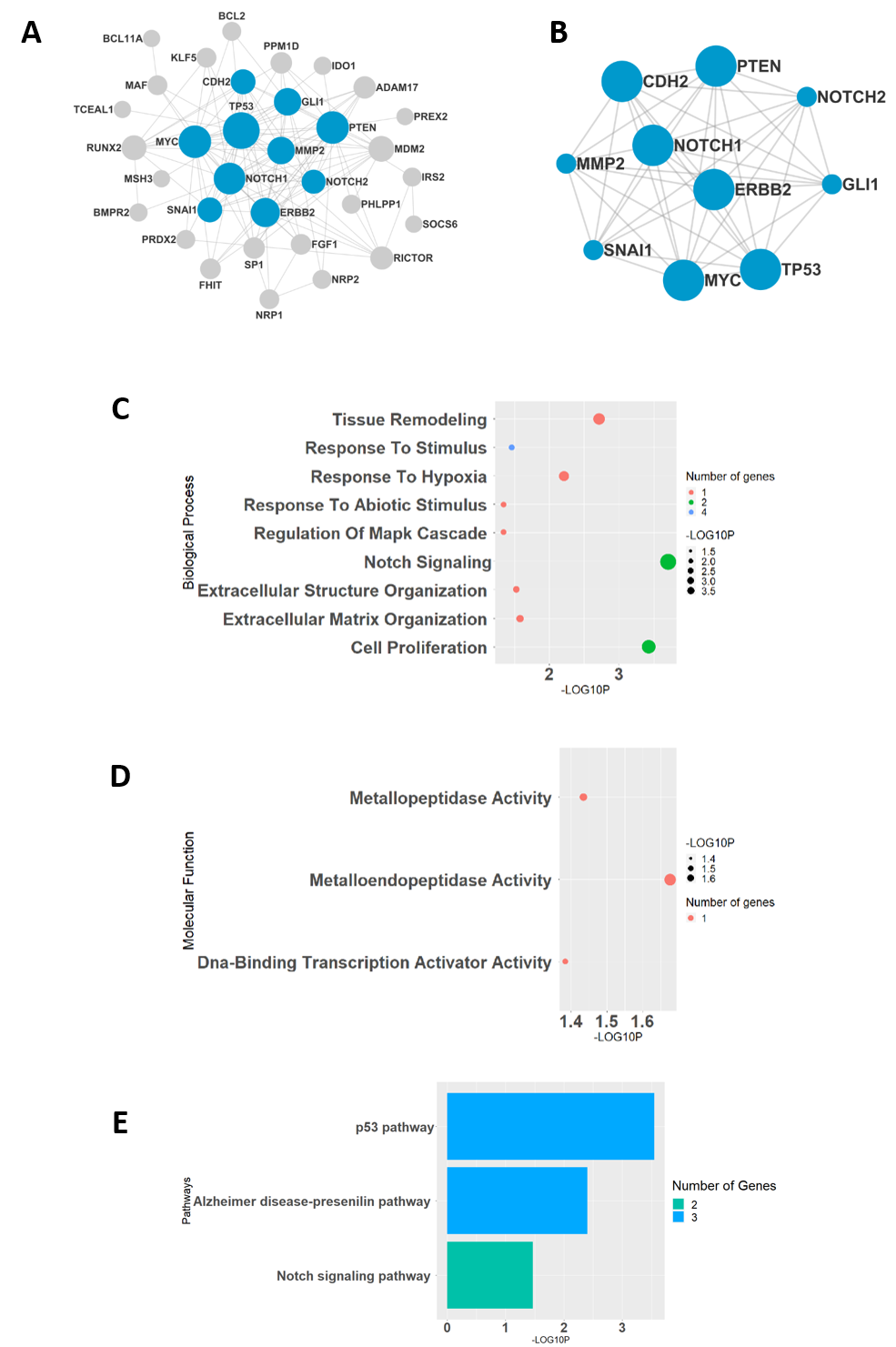


**Supplementary Figure 5**. (A) The Protein Protein Interaction Network (PPIN), in lung cancer. (B) The clusters extracted from the Protein Protein Interaction Network (PPIN), in lung cancer. The size of the nodes are distributed according to the number of in and out degrees. (C-E) : The top most biological process(C), molecular function(D) and pathways(E), in lung cancer. The graph has been plotted on the basis of the significant p values (<=0.05), taken as logarithm of p value (–log10P). The size of the dot is represented by the logarithm of the p value and the color represents the number of genes involved in a particular process.

**
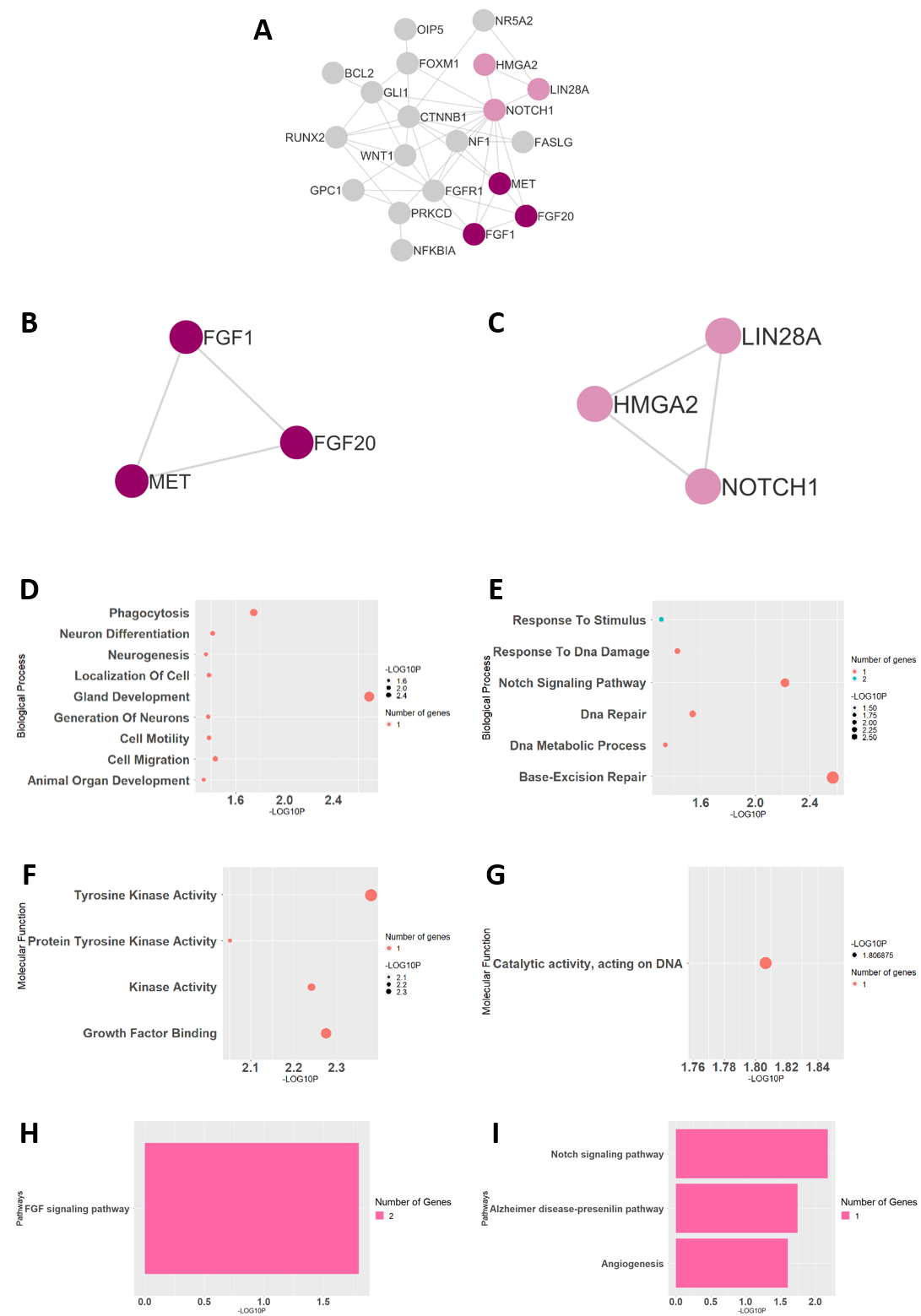
**

**Supplementary Figure 6**. (A) The Protein Protein Interaction Network (PPIN), in breast cancer. (B-C) The clusters (B) cluster 1 and (C) cluster 2, extracted from the Protein Protein Interaction Network (PPIN), in breast cancer. The size of the nodes are distributed according to the number of in and out degrees. (D-I) The top 10 biological process, molecular function and pathways in breast cancer, for cluster 1 (D,F,H), cluster 2 (E,G,I), respectively. The graph has been plotted on the basis of the significant p values (<=0.05), taken as logarithm of p value (–log10P). The size of the dot is represented by the logarithm of the p value and the color represents the number of genes involved in a particular process.


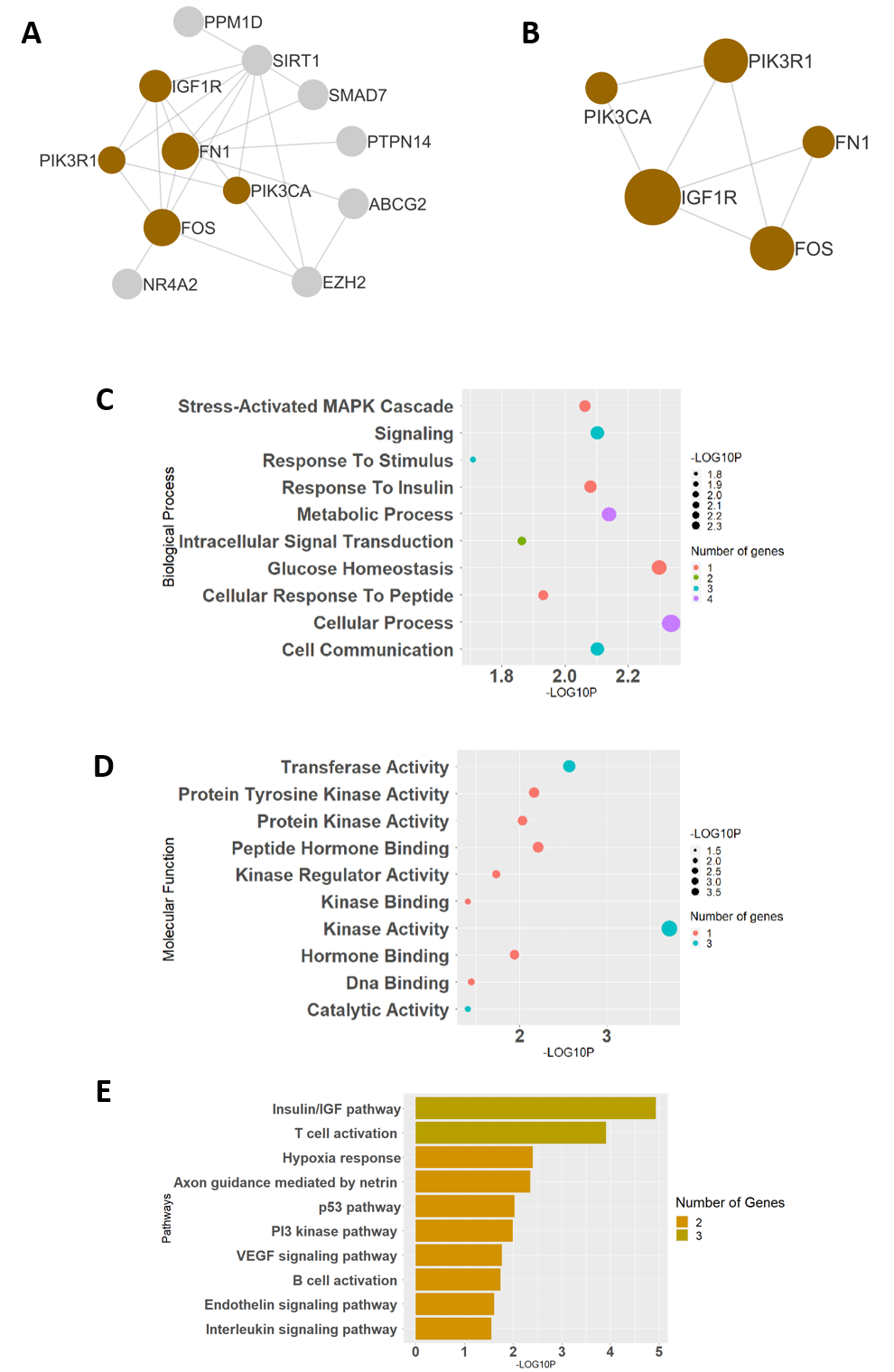


**Supplementary Figure 7**. (A) The Protein Protein Interaction Network (PPIN), in pancreatic cancer. (B) The cluster extracted from the Protein Protein Interaction Network (PPIN), in pancreatic cancer. The size of the nodes are distributed according to the number of in and out degrees. (C-E) The top most biological process(C), molecular function(D) and pathways(E), in pancreatic cancer. The graph has been plotted on the basis of the significant p values (<=0.05), taken as logarithm of p value (–log10P). The size of the dot is represented by the logarithm of the p value and the color represents the number of genes involved in a particular process.


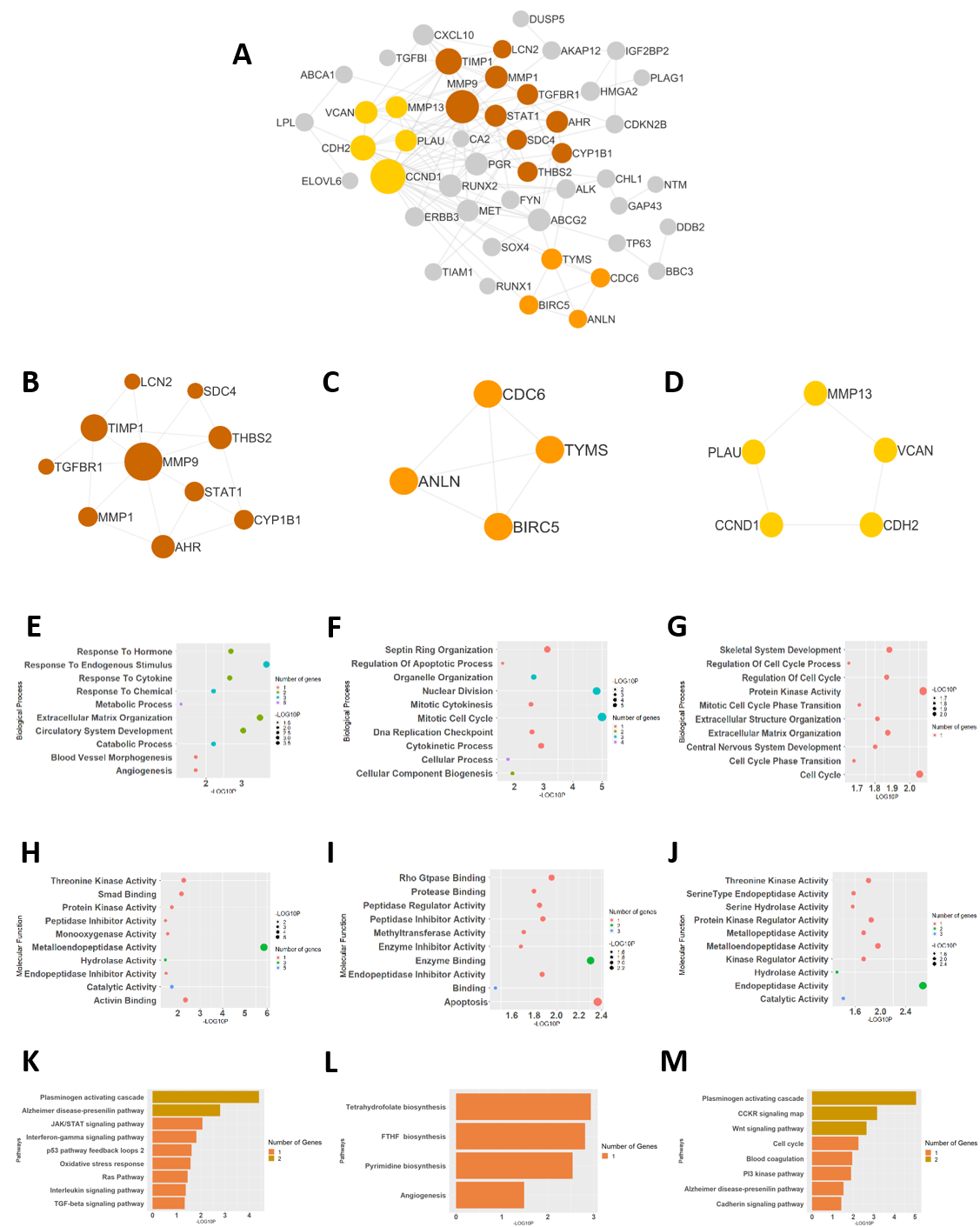


**Supplementary Figure 8**. (A) The Protein Protein Interaction Network (PPIN), in thyroid cancer. (B-D) The clusters (B) cluster 1 (C) cluster 2 and (D) cluster 3, extracted from the Protein Protein Interaction Network (PPIN), in thyroid cancer. The size of the nodes are distributed according to the number of in and out degrees. (E-M) The top 10 biological process, molecular function and pathways in thyroid cancer, for cluster 1 (E,H,K), cluster 2 (F,I,L), and cluster 3 (G,J,M), respectively. The graph has been plotted on the basis of the significant p values (<=0.05), taken as logarithm of p value (–log10P). The size of the dot is represented by the logarithm of the p value and the color represents the number of genes involved in a particular process.


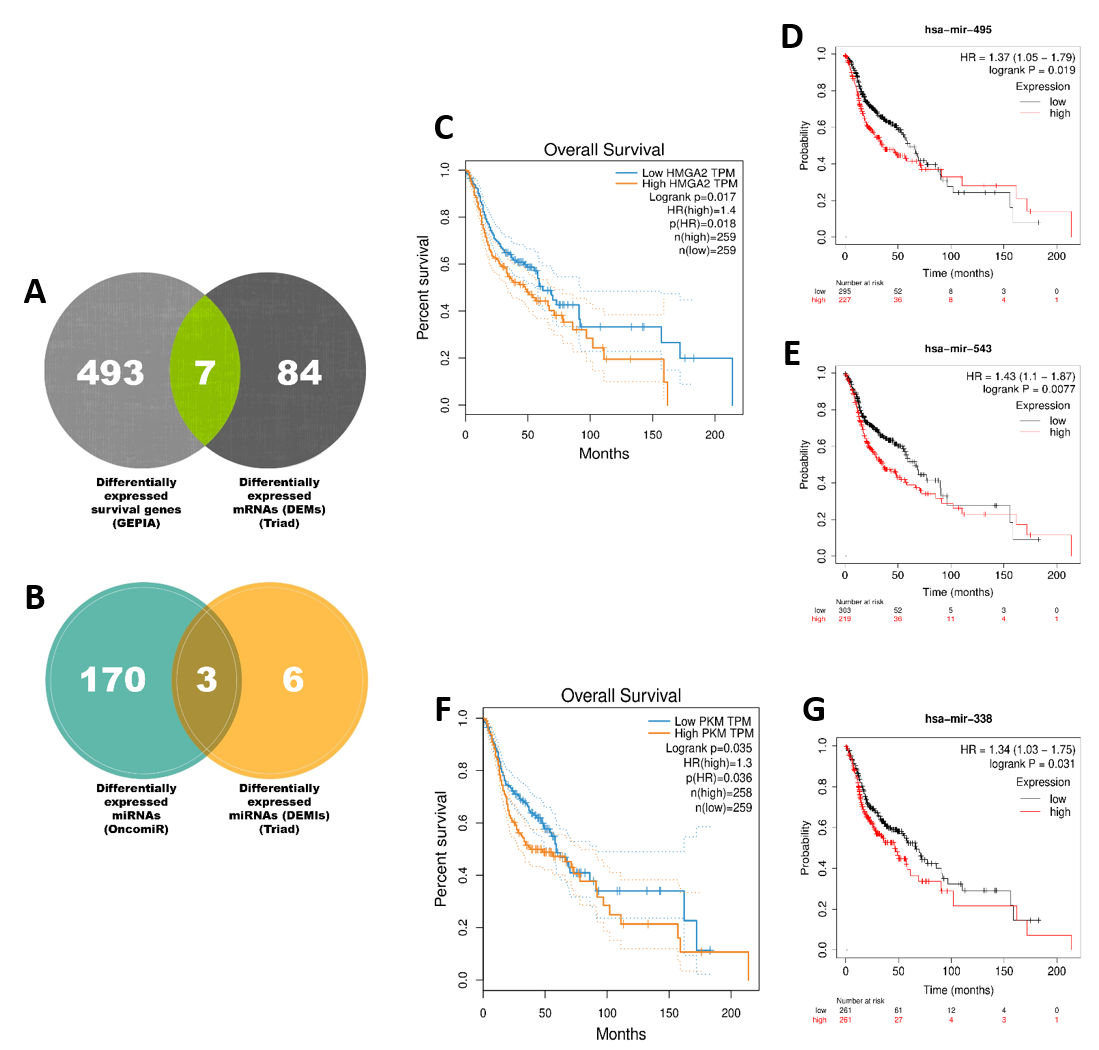


**Supplementary Figure 9**. (A) The venn diagram showing the common genes between the differentially expressed survival genes from GEPIA and the differentially expressed mRNAs from the triad, in HNSCC. (B) The venn diagram showing the common micro RNAs between the differentially expressed survival miRNAs from OncomiR and the differentially expressed miRNAs from the triad, in HNSCC. The survival plots for HMGA2 (C), and its corresponding miRNAs, hsa-miR-495 (D), hsa-miR-543 (E), and for PKM (F) and its corresponding miRNA hsa-miR-338-5p (G). The upregulation of HMGA2 and PKM, and their interacting miRNAs show their role in the survival of the patients. Both the gene and miRNAs decrease patient survival, in HNSCC.


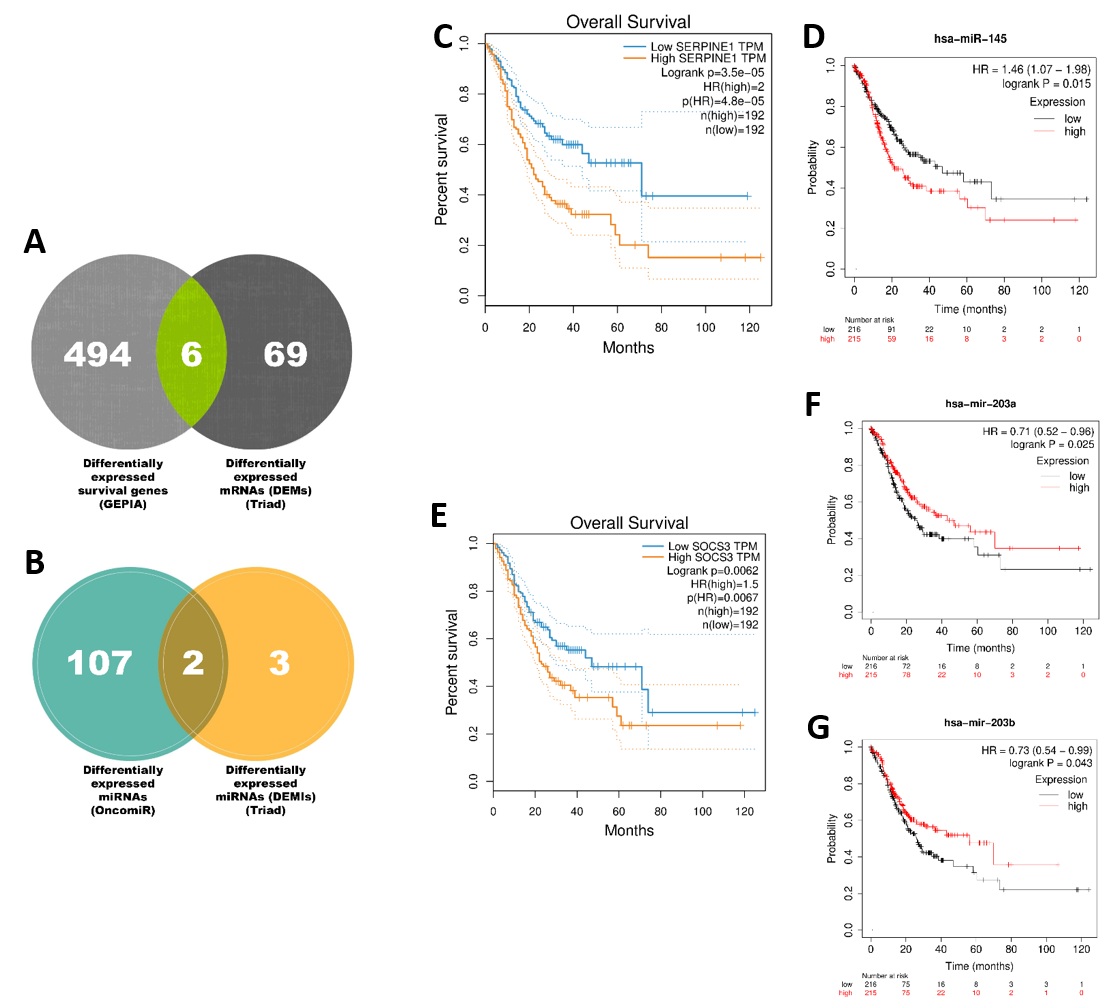


**Supplementary Figure 10.** (A) The venn diagram showing the common genes between the differentially expressed survival genes from GEPIA and the differentially expressed mRNAs from the triad, in gastric cancer. (B) The venn diagram showing the common micro RNAs between the differentially expressed survival miRNAs from OncomiR and the differentially expressed miRNAs from the triad, in gastric cancer. (C-G) : The survival plots for SERPINE1 (C), and its corresponding miRNAs, hsa-miR-145 (D) and for SOCS3 (E) and its corresponding miRNA hsa-miR-203a (F), hsa-miR-203a (G). The upregulation of SERPINE1 and PKM, and their interacting miRNAs show their role in the survival of the patients. Both the gene and miRNAs decrease patient survival.


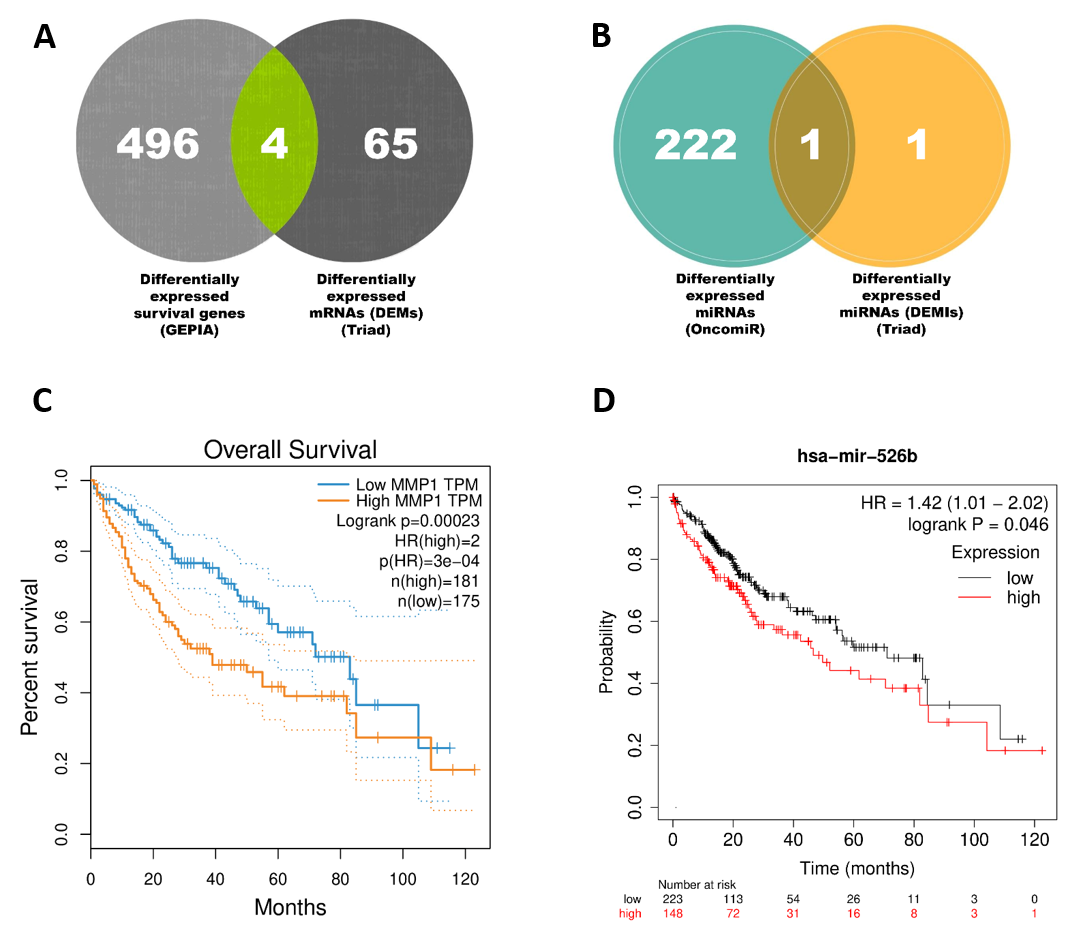


**Supplementary Figure 11**. (A) The venn diagram showing the common genes between the differentially expressed survival genes from GEPIA and the differentially expressed mRNAs from the triad, in liver cancer. (B) The venn diagram showing the common micro RNAs between the differentially expressed survival miRNAs from OncomiR and the differentially expressed miRNAs from the triad, in liver cancer. (C-D) The survival plots for MMP1 (C), and its corresponding miRNA, hsa-miR-526b (D). The upregulation of MMP1, and its interacting miRNA show their role in the survival of the patients. Both the gene and miRNAs decrease patient survival.


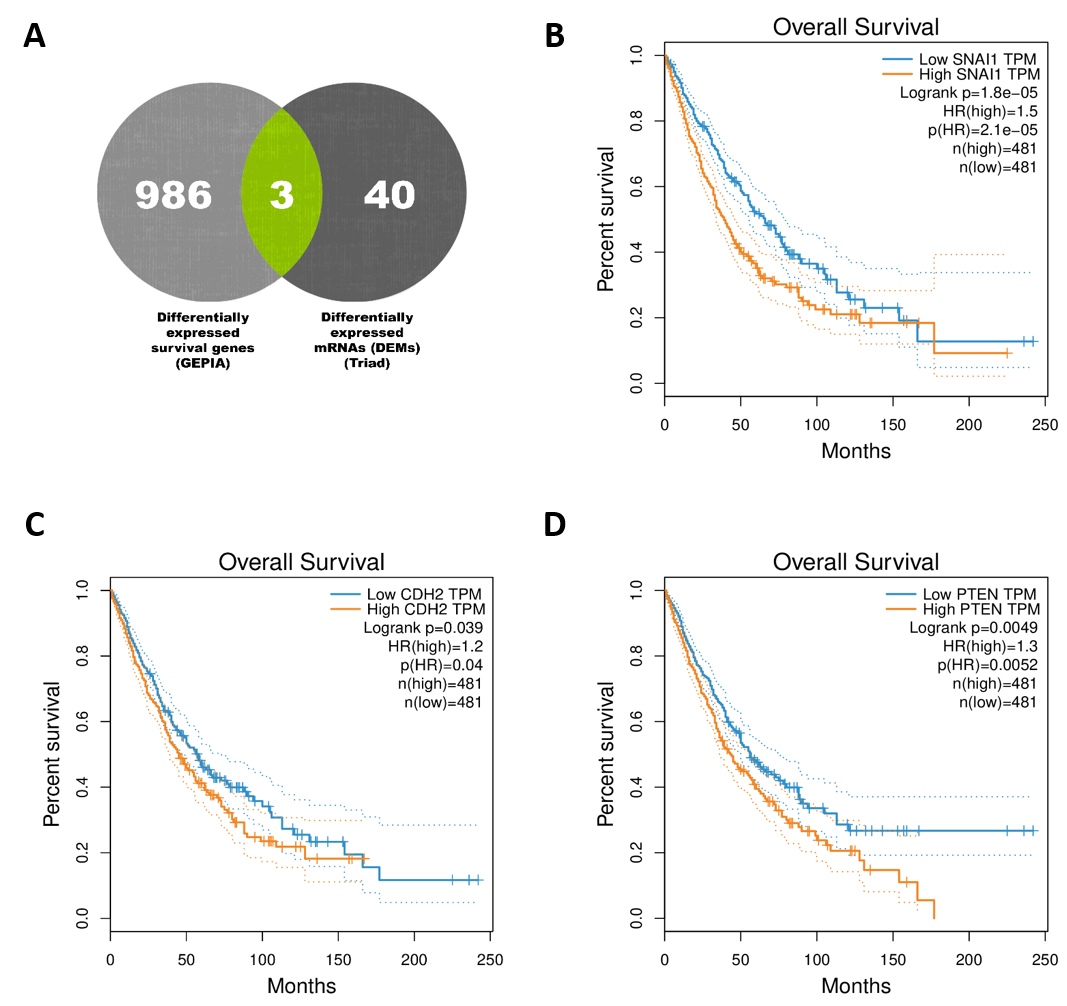


**Supplementary Figure 12**. (A) The venn diagram showing the common genes between the differentially expressed survival genes from GEPIA and the differentially expressed mRNAs from the triad, in lung cancer. (B-D) The survival plots for SNAI1 (B), CDH2 (C), PTEN (D).


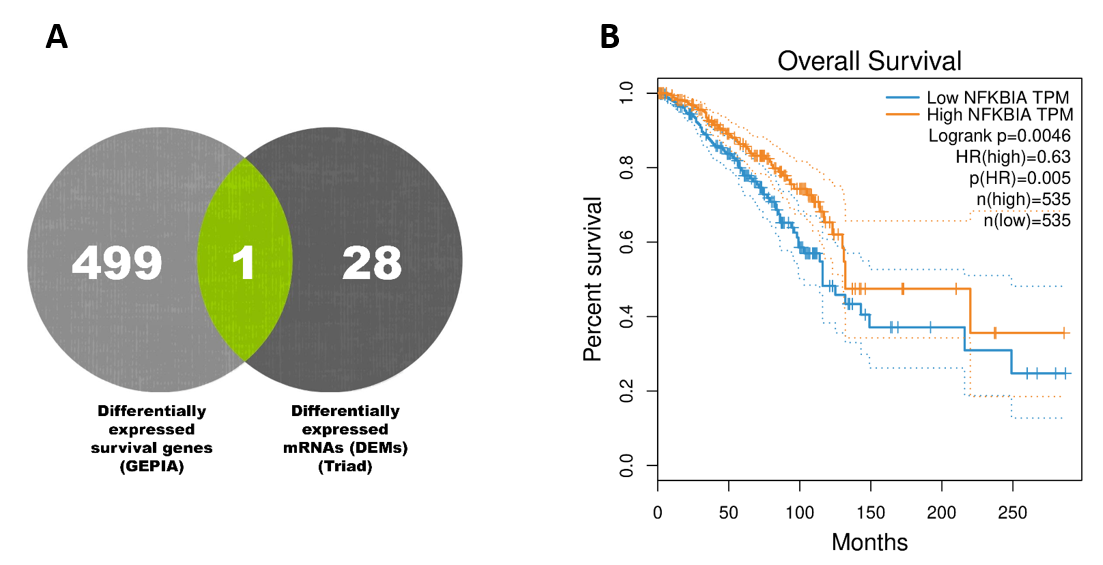


**Supplementary Figure 13**. (A) The venn diagram showing the common genes between the differentially expressed survival genes from GEPIA and the differentially expressed mRNAs from the triad, in breast cancer. (B) The survival plot for NFKBIA.
